# Supplementary material for: Composition of Prokaryotic and Eukaryotic Microbial Communities in Waters around the Florida Reef Tract
Source: Microorganisms. 2021 May 21;9(6):1120. doi: 10.3390/microorganisms9061120 (PMC8224282; doi:10.3390/microorganisms9061120)
Supplement: Supplementary file 1 [file microorganisms-09-01120-s001.zip › Table S2.pdf]

Table S2: Detrended Correspondence Analysis (DCA) of 16 rRNA gene dataset with environmental parameters.

	DCA1	DCA2	r2	Pr	Significance
Latitude	0.72802	0.68556	0.1110	0.565435	
LONDEC	0.40149	0.91586	0.3116	0.144855	
NOX	0.33371	-0.94268	0.2729	0.178821	
NO3	0.32933	-0.94421	0.2816	0.169830	
NO2	0.36519	-0.93093	0.2132	0.283716	
NH4	-0.04412	-0.99903	0.0981	0.604396	
TN	0.15685	-0.98762	0.3098	0.148851	
DIN	0.13993	-0.99016	0.1618	0.422577	
TON	0.15893	-0.98729	0.3252	0.134865	
TP	-0.09116	-0.99584	0.3381	0.098901	
SRP	0.60797	0.79396	0.0873	0.658342	
CHLA	-0.21371	-0.97690	0.1563	0.446553	
**TOC**	**0.49045**	**-0.87147**	**0.7774**	**0.001998**	**Medium**
SiO2	0.95937	-0.28214	0.3119	0.168831	
TURB	-0.03857	-0.99926	0.2207	0.258741	
SAL	-0.26217	0.96502	0.3414	0.129870	
**TEMP**	**0.20170**	**0.97945**	**0.5459**	**0.018981**	**Moderate**
**DO**	**-0.14473**	**-0.98947**	**0.6195**	**0.008991**	**Medium**
Kd	0.26936	-0.96304	0.3187	0.136863	
TN.TP	0.65233	0.75793	0.1532	0.370629	
N.P	0.94429	-0.32910	0.3388	0.114885	
DIN.TP	0.63676	-0.77106	0.1773	0.346653	
Si.DIN	0.52856	0.84890	0.0436	0.786214	
DSIGT	-0.99375	-0.11162	0.1873	0.347652	
Depth	-0.88514	0.46532	0.4097	0.051948	
