# Supplementary material for: Composition of Prokaryotic and Eukaryotic Microbial Communities in Waters around the Florida Reef Tract
Source: Microorganisms. 2021 May 21;9(6):1120. doi: 10.3390/microorganisms9061120 (PMC8224282; doi:10.3390/microorganisms9061120)
Supplement: Supplementary file 1 [file microorganisms-09-01120-s001.zip › Table S3.pdf]

Table S3: Detrended Correspondence Analysis (DCA) of 18 rRNA gene dataset with environmental parameters.

	DCA1	DCA2	r2	Pr	Significance
LAT	-0.83354	0.55246	0.0793	0.667333	
LONG	-0.54720	0.83700	0.3783	0.089910	
NOX	-0.33778	-0.94122	0.3392	0.100899	
NO3	-0.34040	-0.94028	0.3543	0.093906	
NO2	-0.31782	-0.94815	0.2410	0.212787	
NH4	0.38313	-0.92370	0.1211	0.510490	
TN	-0.34662	-0.93800	0.3300	0.129870	
DIN	0.00204	-1.00000	0.1956	0.316683	
TON	-0.38598	-0.92251	0.3455	0.125874	
TP	0.28141	-0.95959	0.3404	0.133866	
SRP	-0.78145	0.62396	0.1621	0.402597	
CHLA	0.67173	-0.74080	0.2079	0.285714	
**TOC**	**-0.59761**	**-0.80179**	**0.6532**	**0.000999**	**High**
SiO2	-0.87916	-0.47653	0.1808	0.379620	
TURB	0.29715	-0.95483	0.2618	0.193806	
SAL	-0.00715	0.99997	0.3202	0.132867	
**TEMP**	**-0.25898**	**0.96588**	**0.4617**	**0.047952**	**Moderate**
**DO**	**0.21156**	**-0.97736**	**0.5536**	**0.016983**	**Moderate**
Kd	-0.02802	-0.99961	0.2514	0.224775	
TN.TP	-0.99290	0.11896	0.2991	0.179820	
N.P	-0.92224	-0.38661	0.2679	0.188811	
DIN.TP	-0.51569	-0.85677	0.1961	0.303696	
Si.DIN	-0.43706	0.89943	0.0470	0.776224	
DSIGT	0.91928	0.39361	0.2021	0.315684	
Depth	0.94645	0.32286	0.3337	0.14285	
